# Nicotinamide Exacerbates Hypoxemia in Ventilator-Induced Lung Injury Independent of Neutrophil Infiltration

**DOI:** 10.1371/journal.pone.0123460

**Published:** 2015-04-13

**Authors:** Heather D. Jones, Jeena Yoo, Timothy R. Crother, Pierre Kyme, Anat Ben-Shlomo, Ramtin Khalafi, Ching W. Tseng, William C. Parks, Moshe Arditi, George Y. Liu, Kenichi Shimada

**Affiliations:** 1 Division of Pulmonary and Critical Care Medicine, Cedars-Sinai Medical Center, Los Angeles, CA, United States of America; 2 Division of Pediatric Infectious Diseases and Immunology, Cedars-Sinai Medical Center, Los Angeles, CA, United States of America; 3 Infectious and Immunologic Diseases Research Center, Cedars-Sinai Medical Center, Los Angeles, CA, United States of America; 4 Pituitary Center, Department of Medicine, Cedars Sinai Medical Center, Los Angeles, CA, United States of America; Section of Pulmonary and Critical Care Medicine, UNITED STATES

## Abstract

**Background:**

Ventilator-induced lung injury is a form of acute lung injury that develops in critically ill patients on mechanical ventilation and has a high degree of mortality. Nicotinamide phosphoribosyltransferase is an enzyme that is highly upregulated in ventilator-induced lung injury and exacerbates the injury when given exogenously. Nicotinamide (vitamin B3) directly inhibits downstream pathways activated by Nicotinamide phosphoribosyltransferase and is protective in other models of acute lung injury.

**Methods:**

We administered nicotinamide i.p. to mice undergoing mechanical ventilation with high tidal volumes to study the effects of nicotinamide on ventilator-induced lung injury. Measures of injury included oxygen saturations and bronchoalveolar lavage neutrophil counts, protein, and cytokine levels. We also measured expression of nicotinamide phosophoribosyltransferase, and its downstream effectors *Sirt1* and *Cebpa*, *Cebpb*, *Cebpe*. We assessed the effect of nicotinamide on the production of nitric oxide during ventilator-induced lung injury. We also studied the effects of ventilator-induced lung injury in mice deficient in C/EBPε.

**Results:**

Nicotinamide treatment significantly inhibited neutrophil infiltration into the lungs during ventilator-induced lung injury, but did not affect protein leakage or cytokine production. Surprisingly, mice treated with nicotinamide developed significantly worse hypoxemia during mechanical ventilation. This effect was not linked to increases in nitric oxide production or alterations in expression of Nicotinamide phosphoribosyl transferase, *Sirt1*, or *Cebpa* and *Cebpb*. *Cebpe* mRNA levels were decreased with either nicotinamide treatment or mechanical ventilation, but mice lacking C/EBPε developed the same degree of hypoxemia and ventilator-induced lung injury as wild-type mice.

**Conclusions:**

Nicotinamide treatment during VILI inhibits neutrophil infiltration of the lungs consistent with a strong anti-inflammatory effect, but paradoxically also leads to the development of significant hypoxemia. These findings suggest that pulmonary neutrophilia is not linked to hypoxemia in ventilator-induced lung injury, and that nicotinamide exacerbates hypoxemia during VILI.

## Introduction

Acute respiratory distress syndrome (ARDS) is a clinical syndrome characterized by an acute onset, bilateral infiltrates on chest x-ray, and profound hypoxemia [1]. Even with best supportive care, the mortality rate for this disease is about 25% [2]. Many patients who develop ARDS require mechanical ventilation, and unfortunately this intervention can precipitate or worsen disease progression [3]. The pressures and volumes used during positive-pressure mechanical ventilation of patients in the ICU can cause an acute lung injury termed ventilator-induced lung injury (VILI). Because of its direct clinical impact, VILI is an area of intense research [3–12].

One particularly active aspect of current research into the mechanisms of VILI focuses on the role of the enzyme nicotinamide phosphoribosyltransferase (NAMPT; also known as Pre-B cell Enhancing Factor). NAMPT is markedly upregulated in VILI and experimental studies indicate that it has diverse roles in augmenting acute lung injury by promoting alveolar permeability [13, 14], promoting neutrophil influx [7], inhibiting neutrophil apoptosis [15], and increasing oxidative stress [16]. Furthermore, intratracheal administration of recombinant NAMPT augments acute lung injury, whereas mice given FK866, a specific noncompetitive inhibitor of NAMPT, or lacking one allele of *Nampt* are protected from VILI [7, 17, 18]. Therefore, NAMPT is upregulated by VILI and aggravates acute lung injury, and its inhibition improves outcomes. The mechanisms by which NAMPT exerts these detrimental effects in VILI are unclear and understanding of the downstream pathways affected by NAMPT will be important for understanding the pathogenesis of acute lung injury.

NAMPT is a pleiotrophic enzyme with several different activities [19], but it is best characterized as the rate-limiting enzyme in the pathway that generates nicotinamide adenine dinucleotide (NAD+) [20]. NAD+ is a coenzyme for four families of enzymes: ADP-ribose cyclases, mono-ADP-ribosyltranferases, poly-ADP ribosyltransferases (PARPs) and the sirtuins, or type III histone deacetylases [21, 22], and some of these enzymes have been implicated in acute lung injury models. [23–30]. Nicotinamide (NAM), also known as vitamin B3, plays a dual role in the NAMPT/NAD+ pathway: NAM is the substrate for NAMPT and therefore the precursor of NAD+, but NAM also directly inhibits all NAD+-dependent enzymes by competing for the NAD+ binding site on these molecules [31]. Therefore, if NAMPT’s pro-inflammatory and injurious effects in VILI are related to generation of NAD+, one might predict that NAM administration could either exacerbate the proinflammatory effects of NAMPT by increasing production of NAD+, or ameliorate NAMPT’s effects by inhibiting downstream NAD+-dependent enzymes. Experimental data has suggested that it is the latter that occurs *in vivo*, because NAM administration is anti-inflammatory in multiple models of inflammation [32–35] including models of acute lung injury. For example, niacin (the oral form of NAM) attenuated pro-inflammatory cytokine production in the lung in both bleomycin-induced [36] and sepsis-induced [37] acute lung injury, and improved survival in the sepsis model. NAM also decreased lung edema and damage in both ischemia/reperfusion [38] and LPS-induced [39, 40] acute lung injury models. We hypothesized that NAM would confer similar beneficial and anti-inflammatory effects in VILI. We found that NAM administration inhibited neutrophil infiltration into the lungs in our mouse model of VILI, consistent with a strong anti-inflammatory effect. To our surprise, however, NAM administration significantly worsened oxygenation. These findings suggest that: 1) neutrophil infiltration of the lungs is disassociated from the development of hypoxemia during VILI; and 2) NAM exacerbates hypoxemia in VILI through an as-yet undefined mechanism.

## Materials and Methods

### Mice

*Cebpe* ^*–/—*^ mice were provided by Dr. H. Phillip Koeffler (Cedars-Sinai Medical Center, Los Angeles, CA). Eight to 10 week old, sex matched 129/SvEv mice and male C57BL/6 mice were obtained from Jackson Laboratories (Bar Harbor, ME).

### Intubation, Mechanical Ventilation, and NAM administration

Mice to be mechanically ventilated were anesthetized with intraperitoneal injections of a mix of ketamine (Vedco Inc., Saint Joseph, MO) and dexmedetomidine (Pfizer, Irvine, CA) (75 mg/kg and 0.5 mg/kg respectively). Mice were orotracheally intubated and ventilated using an Inspira volume-controlled small animal ventilator (Harvard Apparatus, Holliston, MA) with a tidal volume of 20 ml/kg and a respiratory rate of 70 breaths/min with zero positive end-expiratory pressure. NAM (Sigma Aldrich, St. Louis, MO) or an equivalent volume of PBS were administered i.p. after 1 h of mechanical ventilation (MV), and MV was continued for a total of 6 h. PBS (500 μl, s.c.) was administered to all mice undergoing MV at 4 h of MV. Ketamine (50 mg/kg) was administered s.c. as needed (usually every 2–3 h). Mice were kept warm on a heating pad (38°C) (Hallowell EMC, Pittsfield, MA). Non-ventilated control mice received i.p. PBS or NAM at the same time as ventilated mice received PBS or NAM, and remained in their cages until the end of MV.

### Pulse Oximetry

Arterial oxygen saturations were measured in MV mice using a MouseOX pulse oximeter (STARR Life Sciences, Oakmont, PA).

### Serum and Bronchoalveolar Lavage (BAL)

After euthanasia with isofluorane overdose, the abdomen was opened, and peripheral blood was collected in heparinized syringes from the inferior vena cava and centrifuged to obtain serum. The trachea was exposed and cannulated with a 22 G IV cannula. 0.5 ml of PBS with 2 mM EDTA was instilled and aspirated two times. Cells were separated from supernatant, and the total number of cells was determined using a hemoctyometer. Slides were then prepared from cell suspensions and stained with Diff-Quick (Fisher Scientific, Waltham, MA). A differential count was performed on 150 cells per animal, and expressed as an absolute number or percentage of total cells recovered. Serum and BAL supernatants were stored at -80°C for use in ELISAs and for total protein measurements. Lungs were perfused using 2 ml of PBS via injection into the right ventricle, and then were removed. The left lung was separated, placed in RNAlater buffer, and stored at 4°C for RNA extraction. The right lung was removed, flash-frozen in liquid nitrogen, and stored at -80°C for further analysis.

### Detection of cytokines, total protein, and lung MPO

The cytokine concentrations in BAL were determined using Mouse IL-1β ELISA (eBioscience, San Diego, CA), and Mouse keratinocyte-derived chemokine (KC) and macrophage inflammatory protein 2 (MIP2) ELISA kits (R&D Systems, Minneapolis, MN). Total protein in serum and BAL samples was determined using the Bio-Rad DC Protein Assay (Bio-Rad, Hercules, CA). Myeloperoxidase concentrations were measured in lung homogenates using an MPO ELISA assay [41]. Two to three wells were used per sample for all assays.

### Quantitative RT-PCR

The left lung was separated and placed in RNAlater buffer (Life Technologies, Grand Island, NY) and stored at 4°C. RNA was extracted with RNeasy Lipid Tissue Mini Kit (QIAGEN, Valencia, CA) and treated with deoxyribonuclease (QIAGEN, Valencia, CA) to eliminate genomic DNA. Two micrograms of purified total RNA was treated with gDNA elimination buffer and then reverse-transcribed into first-strand cDNA using oligo (deoxythymidine) primers, with QuantiTect reverse transcriptase (QIAGEN, Valencia, CA). Primers used for quantitative RT-PCR are murine *Sirt1* (Mm00490758), *Nos2* (Mm00440502), *Nampt* (Mm00451938), *Cebpa* (Mm00514283), *Cebpb* (Mm00843434), *Cebpe* (Mm02030363), and *Tubulin 1B* (Mm02030931) designed and purchased from Applied Biosystems. A 40-cycle PCR was carried out at 60°C annealing temperature in a MicroAmp Optical 96-well plate in BioRad iQ5 real-time PCR detection system. Amplicons and Taqman murine tubulin control expression assay used as an endogenous reference were detected using the relevant probes tagged with MGB quencher and FAM (carboxyfluorescein) dye (Life Technologies, Grand Island, NY)). Total of 100ng of RNA was loaded into each well. Samples were analyzed in duplicate.

### Histology and Immunostaining

Lungs were perfused-fixed with PBS-buffered formalin through the trachea under 20 cm pressure for 5 min, incubated in fixative at room temperature for 48 hr, and embedded in paraffin. Immunohistochemical detection of rat anti-mouse Ly-6B.2 monoclonal antibody (7/4) (Abd Serotec, Raleigh, NC) was performed on 4-μm tissue sections. Staining was done manually using heat-induced epitope retrieval method in low Citrate 6.0 pH buffer. The staining was performed overnight in 4°C refrigeration at a 1/100 dilution for Ly-6B.2. The following day an anti-rat IgG, HRP linked secondary antibody (Cell Signaling Technology #7077, Beverly, MA) was applied at a dilution of 1/500 for 1 hour at lab room temperature. Tyramide signal amplification was performed using a TSA kit (Life Technologies, Grand Island, NY), and a streptavidin HRP conjugated antibody (Invitrogen) was incubated for 1 hour at lab room temperature at a 1/500 dilution. The staining was visualized using a DAB kit (Vector Labs, Burlingame, CA). Slides were subsequently counterstained with Mayer’s hematoxylin, dehydrated, cleared, and covered. Controls sections were processed with preimmune serum.

### Peripheral Complete Blood Counts and Differentials

Whole blood was collected from the inferior vena cava of euthanized mice using syringes containing EDTA. The blood was immediately analyzed using a Hemavet 950 machine (Drew Scientific Inc.) for complete blood counts and automated differential analysis of white blood cells.

### Detection of C/EBPε protein

Mice were treated with mechanical ventilation and PBS/nicotinamide i.p. as described in Methods (PBS, NAM), and control mice received only PBS or nicotinamide i.p (cPBS, cNAM). All animals were euthanized at the end of the experiment. Lungs were perfused via the right ventricle, and then removed en bloc from the thorax and frozen in liquid nitrogen. Later, frozen lungs were thawed on ice and homogenized in 1 ml of lysis buffer (50 mM HEPES, 100 mM NaCl. 1mM EDTA, 1mM Na3VO4, 10% Glycerol, 0.5% NP-40, protease Inhibitor mix 1%) 10 ug of total lung lysate were loaded per lane for SDS-PAGE, and proteins were transferred to nitrocellulose and probed for C/EBPε using primary antibody against C/EBPε (Santa Cruz Biotechnology Inc.) and corresponding secondary antibody conjugated with horseradish peroxidase (Jackson ImmunoResearch). The blots were visualized using SuperSignal West Pick Chemiluminescent substrate (Thermo Scientific).

### Statistical Analysis

All data were analyzed with the Prism 4.03 statistical program. To compare differences in BAL cell numbers, cytokine levels, and BAL/serum protein ratios, the 2-tailed Student t test (at 95% confidence interval) was used to compare unpaired samples between experimental groups. For experiments involving 3 or more groups, we used 1-way ANOVA with the Tukey post hoc test. For experiments comparing oxygen saturations between groups at different time points, we used the 2-way ANOVA with the Bonferroni post hoc test. A value of p < 0.05 was considered statistically significant.

### Ethics Statement

The study was approved by and all animal experiments were conducted according to the Cedars-Sinai Medical Center Institutional Animal Care and Use Committee guidelines.

## Results

### Nicotinamide decreases BAL polymorphonucleocytes (PMNs) but causes significant hypoxemia in ventilator-induced lung injury (VILI)

**Fig 1:**
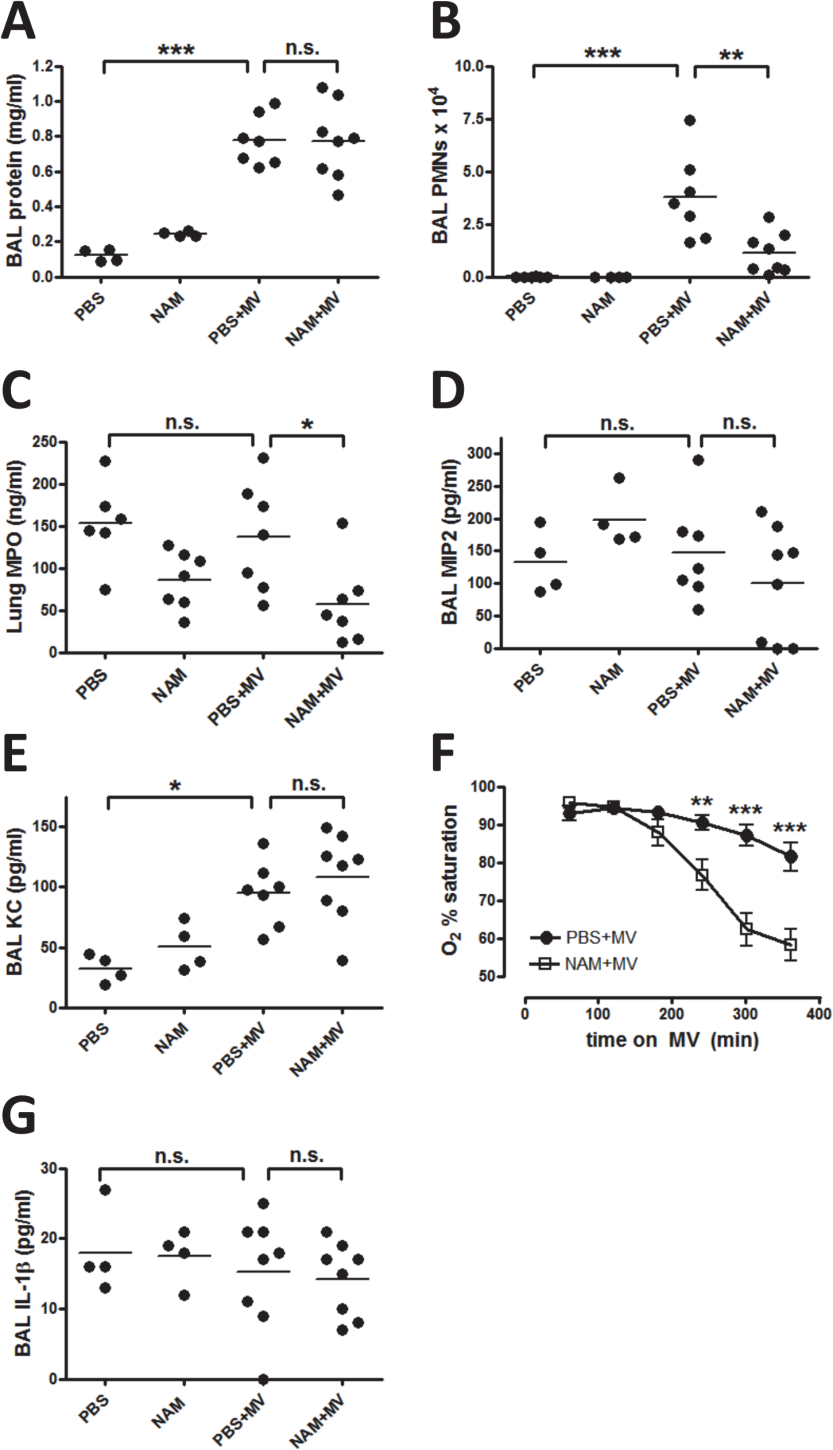
Nicotinamide decreases BAL PMNs but causes significant hypoxemia in VILI. (A) Mice were anesthetized and placed on mechanical ventilation (MV) with tidal volumes of 20 ml/kg and zero PEEP. After 1 hour of mechanical ventilation, mice received intraperitoneal injections of either 400 mg/kg nicotinamide (open squares, NAM+MV) or an equivalent volume of PBS (closed circles, PBS+MV), and mechanical ventilation was continued for a total of 6 hours. All mice were euthanized at the end of 6 hours mechanical ventilation or spontaneous breathing (control mice), which was 5 hours after NAM or PBS administration. Bronchoalveolar lavage (BAL) fluid was assayed for: (A) protein concentration; (B) neutrophils (PMNs); (D and E) chemokines KC and MIP2; and (G) cytokine IL-1 n = 4 /group (non-ventilated controls) and 7-8/group (mechanical ventilation). F) Oxygen saturation was measured each hour. n = 11-12/group. (C) Lung homogenate was assayed via ELISA for MPO content. n = 6-7/group. Data are representative of two (C-E, G) to four (A, B, F) separate experiments. Significance is indicated for comparisons between PBS and PBS+MV, and between PBS+MV and NAM+MV. * p < 0.05, ** p < 0.01, *** p < 0.001.

**Fig 2:**
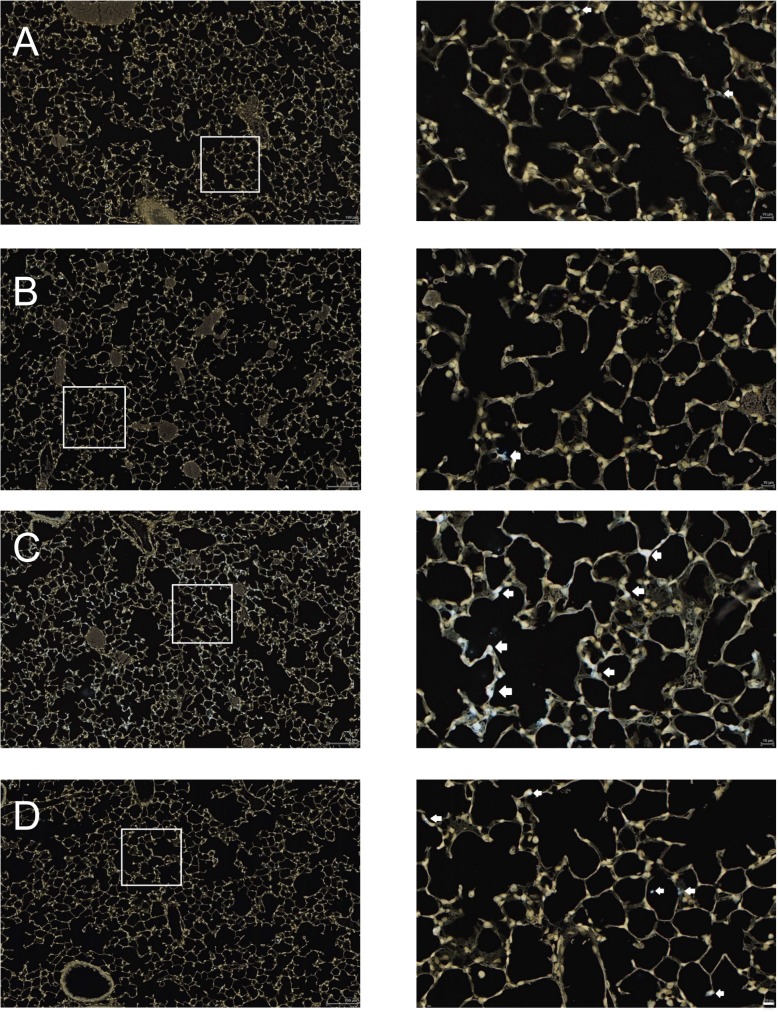
Nicotinamide inhibits neutrophil infiltration of lungs during VILI. Mice were anesthetized and placed on mechanical ventilation (MV) and received i.p. nicotinamide or PBS as described in Fig 1. All mice were euthanized at the end of 6 hours mechanical ventilation or spontaneous breathing (control mice), which was 5 hours after NAM or PBS administration, and lungs were harvested, fixed, and embedded. Sections of lungs were incubated with GR-1, an antibody for murine neutrophils. Stained neutrophils (white arrows) are white in these reverse images of mice treated with: (A) PBS only; (B) Nicotinamide only; (C) PBS and mechanical ventilation; and (D) Nicotinamide and mechanical ventilation. Images are shown at 10x (left panels) and 40x (right panels) magnifications.

We hypothesized that systemic nicotinamide (NAM) would exert anti-inflammatory properties and therefore be protective in a mouse model of ventilator-induced lung injury. Indeed, although NAM did not affect the mechanical ventilation (MV)-mediated increase in BAL protein (Fig 1A), indicating it did not impact the mechanical injury, NAM significantly decreased neutrophil influx in the BAL and lung tissue (Fig 1B and 1C and Fig 2). These findings indicate that alveolar edema in this model was not dependent on alveolar injury due to neutrophil infiltration. BAL concentrations of chemokines KC and MIP2 were not affected by NAM treatment (Fig 1D and 1E], suggesting that NAM’s inhibitory effect on neutrophil infiltration of the lung in VILI was not due to inhibition of chemokine expression. Consistent with earlier findings [42], peripheral blood PMN counts were unchanged by NAM treatment (S1 Fig) suggesting that an overall decrease in PMN counts in the bloodstream is not the cause of decreased PMN infiltration into the lungs.

**Fig 3:**
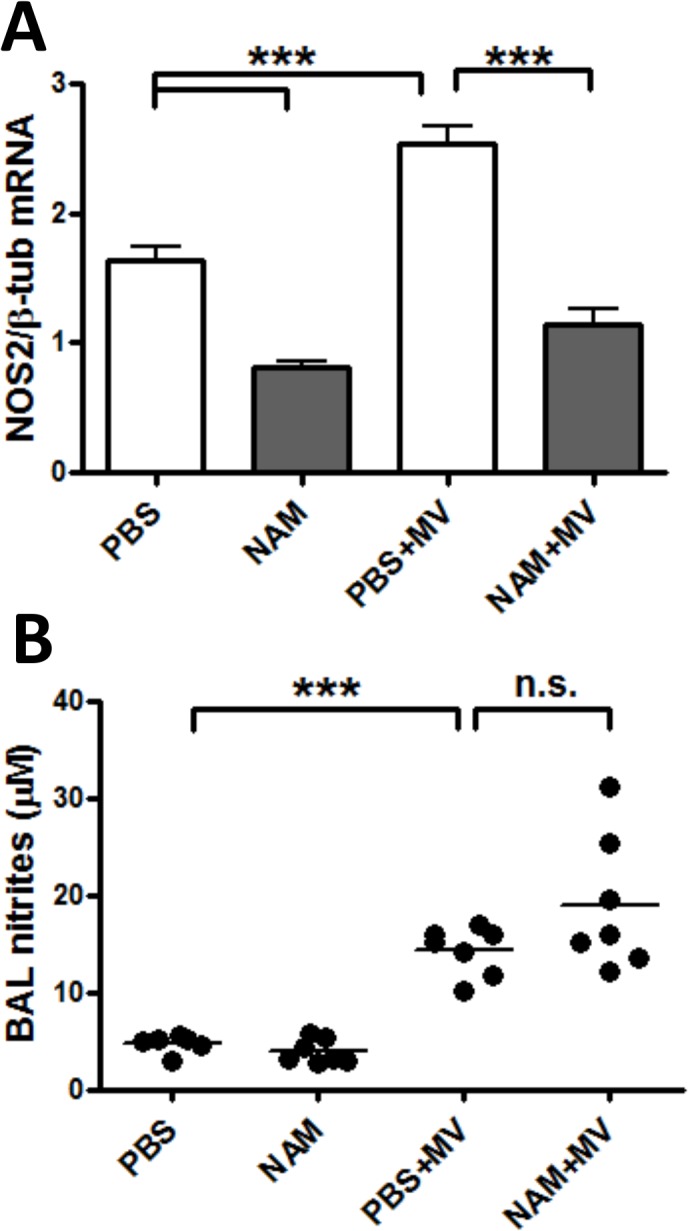
Effects of nicotinamide on hypoxemia and PMNs are dose-dependent. Mice were anesthetized and placed on mechanical ventilation as described in Fig 1. After one hour of mechanical ventilation, mice were injected intraperitoneally with PBS or NAM at doses of 50 mg/kg (NAM50+MV), 200 mg/kg (NAM200+MV) or 400 mg/kg (NAM400+MV). (A) Oxygen saturation was measured each hour. Significance is indicated for comparisons between NAM400 and NAM50 (** p < 0.01, *** p < 0.001) and between NAM400 and NAM200 (^#^ p < 0.05, ^###^ p < 0.001). Mice were euthanized at the end of 6 hours mechanical ventilation, and bronchoalveolar lavage (BAL) fluid was assayed for: (B) protein concentration and (C) neutrophil (PMNs) numbers and percentages of total BAL cells. PBS and NAM400 data are repeated from Fig 1 for comparison with NAM50 and NAM200. Data are representative of two separate experiments for NAM50 and NAM200, and four separate experiments for PBS and NAM400. Significance is indicated for comparisons between PBS and PBS+MV, and between PBS+MV and all NAM+MV doses. * p < 0.05, ** p < 0.01, *** p < 0.001.

Surprisingly, in comparison to PBS-treated mice, NAM-treated mice developed significant, progressive hypoxemia while on mechanical ventilation (Fig 1F). Although reduced neutrophil influx was consistent with our proposed anti-inflammatory effect of NAM, fewer neutrophil likely do not account for the increased hypoxemia with NAM+MV. In LPS+MV-induced ALI, IL-1β caused hypoxemia [43], therefore we measured IL-1β in BAL. However, we did not observed any increase of IL-1β (Fig 1G), suggesting that IL-1β is not responsible for the progressive hypoxemia induced by NAM+MV. In addition, BAL TNF-α and IL-6 were increased with mechanical ventilation, and not affected by NAM (S2 Fig). The responses of neutrophil numbers and oxygen saturations to NAM treatment displayed a dose-response effect, whereas edema did not (Fig 3A–3C). Because the effect is consistent and dose-dependent, the higher NAM dose of 400 mg/kg was used for all subsequent experiments.

### Nicotinamide decreases lung expression of C/EPBε in VILI

**Fig 4:**
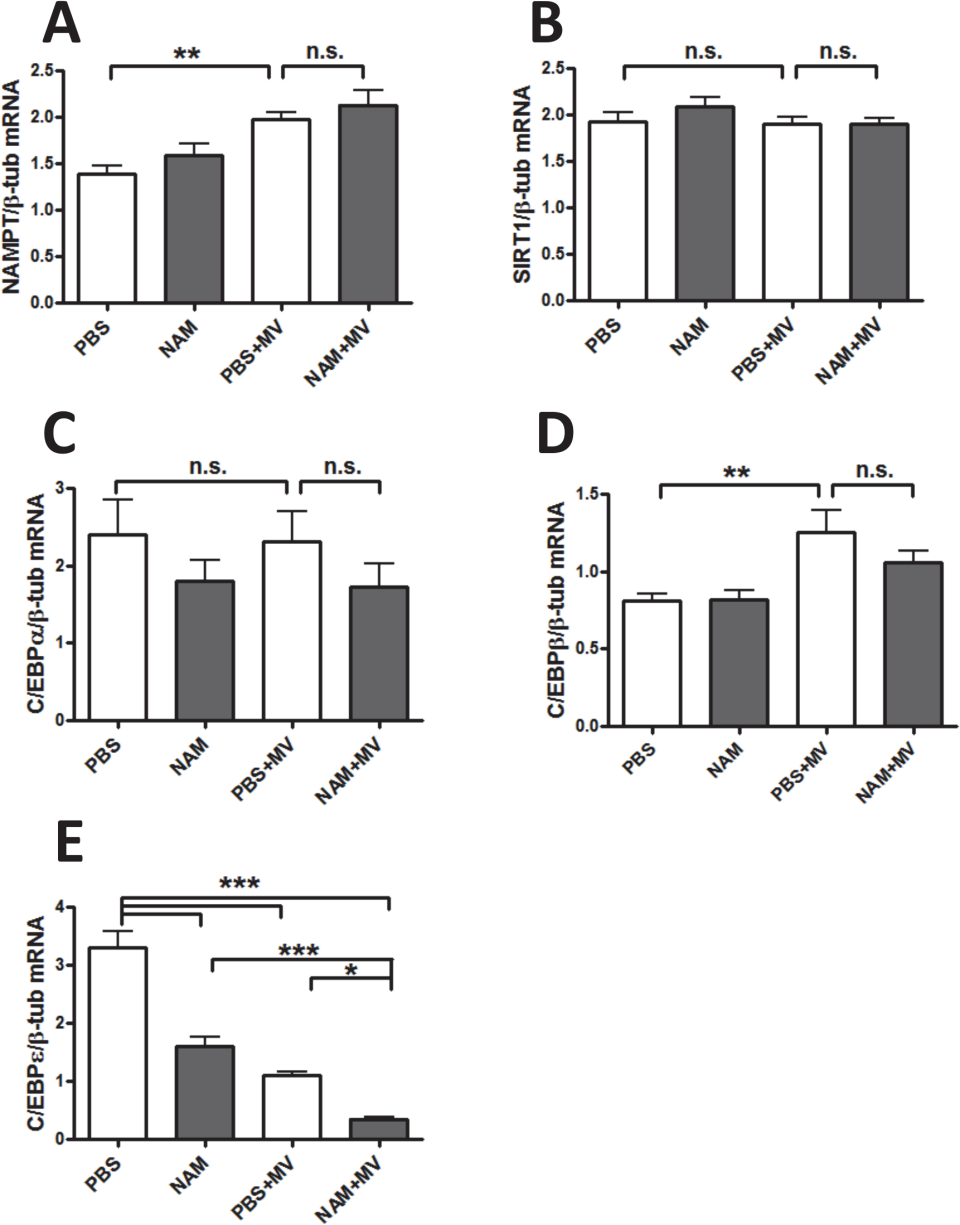
Nicotinamide and mechanical ventilation decrease lung expression of *Cebpe*. Mice were anesthetized and placed on mechanical ventilation as described in Fig 1 and received either PBS (PBS+MV) or nicotinamide at 400 mg/kg (NAM+MV) after 1 hour of mechanical ventilation. Control mice were allowed to continue spontaneously breathing and received PBS or nicotinamide at the same time as mice on mechanical ventilation (PBS, NAM). Mice were euthanized at the end of 6 hours mechanical ventilation, and the left lung was placed in RNeasy buffer and homogenized. RT-PCR was performed using TaqMan primers for (A) *Nampt*; (B) *Sirt1*; (C)*Cebpa*; (D) *Cebpb*; and (E) *Cebpe* Significance is indicated for comparisons between PBS and PBS+MV, and between PBS+MV and NAM+MV unless otherwise shown. * p < 0.05, ** p < 0.01, *** p < 0.001.

NAM, NAMPT, and NAD+ are components of an integrated signaling pathway. We assessed if NAM affected the expression of *Nampt*, the histone deacetylase SIRT1 (which requires NAD+ as a cofactor, and is directly inhibited by NAM), and SIRT1-regulated transcription factors *Cebpa*, *Cebpb*, and *Cebpe*. MV mediated a significant increase in the expression of *Nampt*, as seen by others [7, 44], and *Cebpb*,but did not influence the levels of *Sirt1* nor *Cebpa* mRNA expression (Fig 3). NAM did not affect the basal and MV-regulated levels of these four transcripts. In contrast, expression of *Cebpe* which functions in neutrophil development and function [45], was significantly decreased by either NAM treatment or MV, and the combination of NAM and MV resulted in about a 10-fold reduction in *Cebpe* mRNA levels compared with controls (Fig 4E). C/EBPε protein concentrations in lung tissue (S3 Fig) were variable, and there was no consistent effect of NAM or MV on C/EBPε protein levels.

### *C/EBPε*-deficient mice have decreased lung PMNs but equivalent hypoxemia in VILI

**Fig 5:**
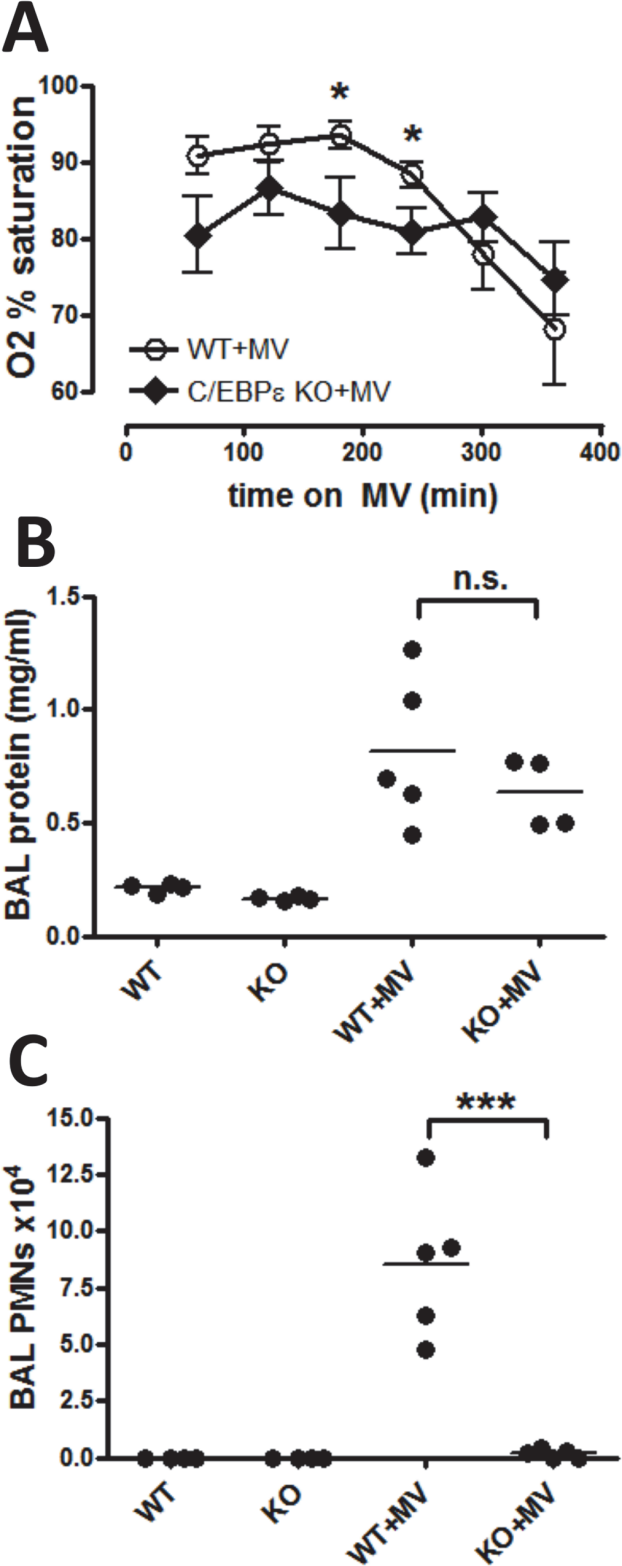
C/EBPε-deficient mice have decreased PMNs but develop equivalent hypoxemia and edema with VILI. (A) Wild-type (WT+MV, open circles) and *Cebpe* knockout (*Cebpe* +MV, closed diamonds) mice were anesthetized and placed on mechanical ventilation (MV) with tidal volumes of 20 ml/kg and zero PEEP for a total of 6 hours. Oxygen saturation was measured each hour for six hours. n = 5/group. (B) Wild type and C/EBPε-deficient mice were anesthetized and placed on MV as described in (A) (WT+MV, +MV); control mice were not anesthetized or mechanically ventilated (WT, KO). All mice were euthanized at the end of 6 hours mechanical ventilation or spontaneous breathing (control mice). Bronchoalveolar lavage (BAL) fluid was assayed for: (B) protein concentration; and (C) neutrophils (PMNs). n = 4 /group (non-ventilated controls) and 5/group (mechanical ventilation). Significance is indicated for comparisons between WT+MV and *Cebpe* KO+MV groups in all graphs. * p <. 05, *** p <. 001.

Because both NAM treatment and MV downregulated *Cebpe* mRNA levels in lung tissue, we assessed if decreased C/EBPε was linked to hypoxemia or inhibition of lung neutrophilia in VILI. Wildtype (WT) and *Cebpe* ^-/-^ mice were placed on mechanical ventilation for 6 h, and oxygen saturations were measured every hour. *Cebpe* ^*-/-*^ mice demonstrated lower oxygen saturations at earlier times (180 and 240 min of MV), but the differences in hypoxemia were not seen at later time points (Fig 5A). These findings suggest that C/EBPε deficiency was not responsible for the hypoxemia observed in mice with NAM treatment on MV. PMN infiltration into the lung with VILI was highly suppressed in *Cebpe* ^*-/-*^ mice (Fig 5C), consistent with established *Cebpe* ^-/-^ phenotypes [46]. BAL protein levels were increased equally in WT and *Cebpe* ^-/-^ mice with MV, again demonstrating a disconnect between PMN infiltration and alveolar leakage in VILI (see Figs 1 and 2). BAL IL-1, MIP2, and KC concentrations were similar between WT and *Cebpe* ^-/-^ mice after MV (data not shown).

### Nicotinamide decreases lung expression of NOS2 in VILI

**Fig 6:**
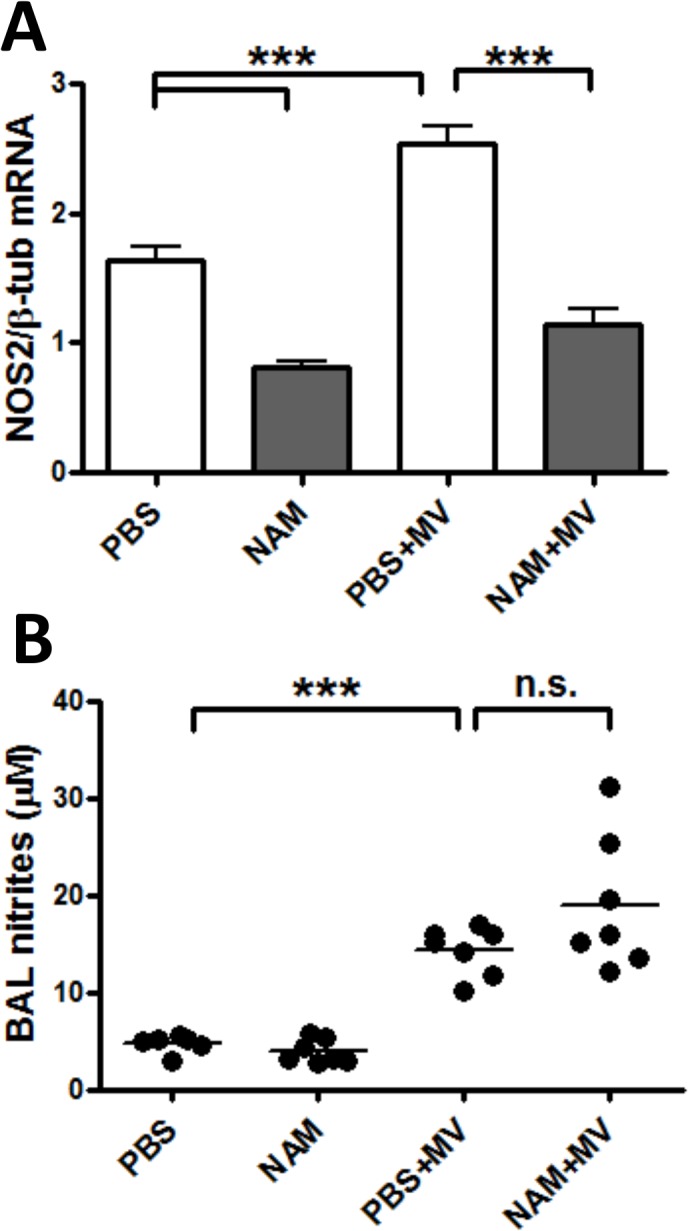
Nicotinamide decreases *Nos2* expression but does not affect BAL nitrites in VILI. Mice were anesthetized and placed on mechanical ventilation as described in Fig 1 and received either PBS (PBS+MV) or nicotinamide at 400 mg/kg (NAM+MV) after 1 hour of mechanical ventilation. Control mice were not anesthetized, and received intraperitoneal NAM or PBS at the same as the mice on mechanical ventilation (PBS, NAM). All mice were sacrificed at the end of 6 hours mechanical ventilation or spontaneous breathing (control mice), which was 5 hours after NAM or PBS administration. (A) RNA was extracted from lungs and RT-PCR was performed using TaqMan primers for *Nos2*. (B) Bronchoalveolar lavage (BAL) fluid was assayed for nitrites. n = 6–7 mice/group. *** p <. 001.

Because nitric oxide levels are increased in the BAL fluid of patients with acute lung injury [47] and because NAM has been reported to inhibit nitric oxide synthase 2 (NOS2) expression [48], we examined the effect of NAM on *Nos2* mRNA levels. *Nos2* expression was increased with MV, and significantly decreased by NAM treatment (Fig 6A). We measured BAL nitrite concentrations and found that MV increased BAL nitrites, as reported [47, 49], but that NAM had no effect on MV-induced increases in BAL nitrites (Fig 6B). Therefore, we conclude that the effects of NAM on hypoxemia in our VILI model cannot be attributed to specific effects on lung NOS2 activity.

## Discussion

The main findings of this research are that nicotinamide treatment during VILI inhibits neutrophil infiltration of the lungs but, paradoxically, also leads to the development of significant hypoxemia. Both PMN inhibition and hypoxemia in response to NAM treatment during VILI occur in a dose-dependent fashion. The observation that PMN infiltration can be distinct from the development of hypoxemia in VILI challenges the assumption that individual measures of acute lung injury can serve as surrogates for the disease process as a whole, and suggests that the mechanisms behind hypoxemia in acute lung injury may be more complicated than previously thought.

We were interested in exploring the effects of NAM in VILI as an inhibitor of NAMPT-related pathways because of the growing body of evidence related to the role of NAMPT in this disease. NAMPT exerts a pro-inflammatory effect in VILI, increasing neutrophils, edema, BAL pro-inflammatory cytokines, and histological damage [7, 14, 50], and anti-NAMPT therapy with specific inhibitors improves these measures [18]. We hypothesized that NAM would be protective in VILI because it is a direct inhibitor of key enzymes that are downstream of and controlled by NAMPT [19–21, 31] and because it has been shown to be strongly anti-inflammatory *in vitro* [32, 33, 48] and *in vivo* in models of inflammatory disease [34], including other models of acute lung injury [35, 40, 51–54]. Indeed, we found a significant and dose-dependent inhibitory effect of NAM treatment during VILI on BAL PMN concentrations. Lung MPO concentrations were measured as a reflection of total lung PMNs, and were also reduced, and this was confirmed on histological examination, ruling out the possibility that NAM treatment merely prevented PMNs from reaching the alveolar space but did not inhibit PMNs from entering the lung interstitium during VILI. BAL cytokines were not affected by NAM treatment, suggesting that NAM’s effect is related to the inhibition of neutrophil transmigration out of the vasculature rather than an inhibition of chemokine production in the lungs.

Our results are in contrast to other work on NAM’s effects on neutrophils. Skokawa et al. showed that NAM administration induced neutrophilic granulopoesis *in vivo* through high intracellular NAMPT and NAD+ levels and subsequent induction of SIRT1 and C/EBP transcription factor activation [20]. Additionally, researchers from our group demonstrated that NAM treatment in a *S*. *aureus* skin infection model strongly enhanced the ability of neutrophils to clear infection due to increased bactericidal activity with NAM treatment, which was dependent on an increased expression of C/EBPε in neutrophils [42]. However, we suggest that NAM’s inhibitory effects on neutrophil transmigration to an area of sterile injury as is seen in the lungs in VILI may be distinct from NAM’s effects on neutrophil maturation and/or neutrophil activity against bacterial infection, and that this differential effect may be an important area for further research.

The finding that NAM inhibited PMN infiltration into the lung during VILI, but at the same time caused progressive and dose-dependent decreases in oxygen saturations, was entirely unexpected. Many models of lung injury measure PMN infiltration, alveolar edema, and inflammatory cytokines as indicators of the degree of lung injury, and improvements in these parameters are taken as evidence of improvement in acute lung injury [55–57]. Oxygen saturation is rarely reported as a measure of an acute lung injury model in mice, although hypoxemia is one of the most important clinical manifestations of acute lung injury. In this paper we found that PMN infiltration, a hallmark of acute lung inflammation and injury, is completely disassociated from hypoxemia. This is difficult to reconcile with prior research that has established a clear role for neutrophils in the development of acute lung injury. Neutrophil depletion has been shown to decrease alveolar edema and inflammatory cytokine concentrations in animal models [58–60], and mechanisms of neutrophil-induced lung injury are well established [61–66], and neutrophil infiltration of the lungs and protein-rich alveolar edema are hallmarks of the development of acute lung injury, both in humans and in animal models [55–57].

However, we recently reported a similar finding in a “two-hit” lipopolysaccharide and MV model of acute lung injury, in which abrogation of IL-1β signaling prevented the development of hypoxemia but did not affect the level of PMN infiltration into the lung or the development of alveolar edema [43]. In that study, we also depleted PMNs from mice using anti-neutrophil antibodies, and found that the development of hypoxemia occurred at the same rate as control mice. It is unclear why hypoxemia is distinct from other measures of acute lung injury; because oxygen saturations are rarely measured in small animals studies of acute lung injury, little is known about this variable in relation to other measures of acute lung injury. One possible explanation for this disconnect could be an alteration in patterns of pulmonary blood flow that leads to increased ventilation-perfusion mismatching. NOS2 is a determinant of pulmonary vascular blood flow [67–69], and nitric oxide production is increased in acute lung injury [47]; we therefore explored whether exogenous NAM could affect *Nos2* expression levels in the lung. Indeed, we found that MV increased *Nos2* expression in the lung, and that NAM administration strongly inhibited this increase. However, although BAL nitrite levels (as a marker of lung NO production) were increased with MV, as has been described in other work [47], NAM treatment during MV did not decrease BAL nitrite levels. It is possible that compartmentalized effects of NAM on *Nos2* transcription may be more important than global effects in the lung that are demonstrated in BAL; for example, if endothelial NOS2 production is important for maintaining ventilation-perfusion matching in VILI, this may not be reflected in BAL nitrites. At this point, the mechanism for NAM-associated hypoxemia in VILI remains unclear. We are actively exploring this question because of the obvious clinical relevance, and because prior work suggests that the NAM/NAMPT/NAD axis is important in VILI [7, 13, 14, 16, 44, 50], and this line of inquiry may help to explain why.

We investigated the impact of MV and NAM treatment on expression of molecules in this axis: the enzyme NAMPT; the histone deacetylase Sirt1 (which is activated by NAMPT and inhibited by NAM); and C/EBPα, β, and ε, which are three transcription factors modulated by Sirt1. We found a marked decrease in *Cebpe* mRNA expression with either MV or NAM treatment, and the effect was additive (Fig 4). To explore this further, we tested the effects of MV in C/EBPε-deficient mice, which have markedly dysfunctional neutrophils due to a lack of C/EBPε. However, although lung neutrophilia was absent in *Cebpe* ^-/-^ mice (Fig 5), hypoxemia developed over time to the same extent in both WT and *Cebpe* ^-/-^ mice on MV. This suggests that inhibition of C/EBPε with NAM treatment was not the cause of progressive hypoxemia in NAM-treated mice. Further investigation will be needed to understand these complex relationships, but the lack of association between PMN infiltration into the lungs and the development of hypoxemia again suggests that these two components of acute lung injury may not be connected.

A caveat to our work is that the relationships between intracellular and extracellular NAMPT and NAD+ are poorly understood. Extracellular NAMPT has been shown to be a robust producer of systemic NAD+ [70], although its physiological role is not clear. Exogenous NAM administration increases intracellular NAD+ concentrations *in vitro* and in human volunteers, suggesting that exogenous NAM can affect the intracellular balance of NAD+ [20, 21]. We did not measure intracellular or extracellular NAD+ concentrations, and so the effects of NAM administration on this parameter in our model of VILI are unknown. It is possible that one concentration of exogenous NAM provides more substrate to drive the formation of NAD+, whereas another concentration of NAM exerts predominantly inhibitory effects on downstream NAD+ dependent enzymes. However, our data demonstrated a consistent dose-dependent effect with increasing NAM doses. The complex relationships between intra- and extracellular NAMPT, NAD+, and NAM in VILI and other diseases will require further work.

In conclusion, we explored the anti-inflammatory effects of nicotinamide administration in a mouse model of VILI and found that although PMN infiltration of the lungs was significantly inhibited by NAM treatment, severe hypoxemia also developed in these mice. This finding is novel and suggests a new area of investigation, particularly in relation to the NAM/NAMPT/NAD+ axis in acute lung injury and the mechanisms of hypoxemia in this disease process. Further research will be directed at determining the role of NAMPT and its downstream effectors specifically in the development of hypoxemia in VILI.

## Supporting Information

S1 Fig Nicotinamide does not affect neutrophil counts in peripheral blood. Mice were anesthetized and placed on mechanical ventilation (MV) with tidal volumes of 20 ml/kg and zero PEEP. After 1 hour of mechanical ventilation, mice received intraperitoneal injections of either 400 mg/kg nicotinamide (NAM+MV) or an equivalent volume of PBS (PBS+MV), and mechanical ventilation was continued for a total of 6 hours; control mice received intraperitoneal NAM or PBS at the same as the mice on mechanical ventilation (PBS, NAM) but were not anesthetized. All mice were euthanized at the end of 6 hours mechanical ventilation or spontaneous breathing (control mice). Whole blood was collected in syringes containing EDTA from the inferior vena cava, and analyzed for complete blood cincluding leukocyte counts and types of leukocytes (automated differential). Differences in neutrophil counts among groups did not reach statistical significance. n = 6-7/group.
(TIF)

S2 Fig Nicotinamide does not affect BAL IL-6 or TNF alpha concentrations during VILI. Mice were anesthetized and placed on mechanical ventilation (MV) with tidal volumes of 20 ml/kg and zero PEEP. After 1 hour of mechanical ventilation, mice received intraperitoneal injections of either 400 mg/kg nicotinamide (NAM+MV) or an equivalent volume of PBS (PBS+MV), and mechanical ventilation was continued for a total of 6 hours; control mice received intraperitoneal NAM or PBS at the same as the mice on mechanical ventilation (PBS, NAM) but were not anesthetized. All mice were euthanized at the end of 6 hours mechanical ventilation or spontaneous breathing (control mice), and bronchoalveolar lavage (BAL) fluid was assayed for: (A) IL-6 and (B) TNF. Differences in IL-6 and TNF concentrations between PBS+MV and NAM+MV groups did not reach statistical significance. N.D. signifies groups for which IL-6 and TNF concentrations were below the limit of detection. n = 6-7/group.
(TIF)

S3 Fig Nicotinamide does not affect lung C/EBPepsilon concentrations. Mice were anesthetized and placed on mechanical ventilation (MV) with tidal volumes of 20 ml/kg and zero PEEP. After 1 hour of mechanical ventilation, mice received intraperitoneal injections of either 400 mg/kg nicotinamide (NAM) or an equivalent volume of PBS (PBS), and mechanical ventilation was continued for a total of 6 hours; control mice received intraperitoneal NAM or PBS at the same as the mice on mechanical ventilation (cPBS, cNAM) but were not anesthetized. All mice were euthanized at the end of 6 hours mechanical ventilation or spontaneous breathing (control mice). Whole lungs were homogenized in lysis buffer and analyzed via Western for C/EBPepsilon protein.
(TIF)
