# Correction: Nicotinamide Exacerbates Hypoxemia in Ventilator-Induced Lung Injury Independent of Neutrophil Infiltration

**DOI:** 10.1371/journal.pone.0128735

**Published:** 2015-05-21

**Authors:** Heather D. Jones, Jeena Yoo, Timothy R. Crother, Pierre Kyme, Anat Ben-Shlomo, Ramtin Khalafi, Ching W. Tseng, William C. Parks, Moshe Arditi, George Y. Liu, Kenichi Shimada

Fig 3 is incorrect. Please view the correct Fig 3 here.

**Fig 3:**
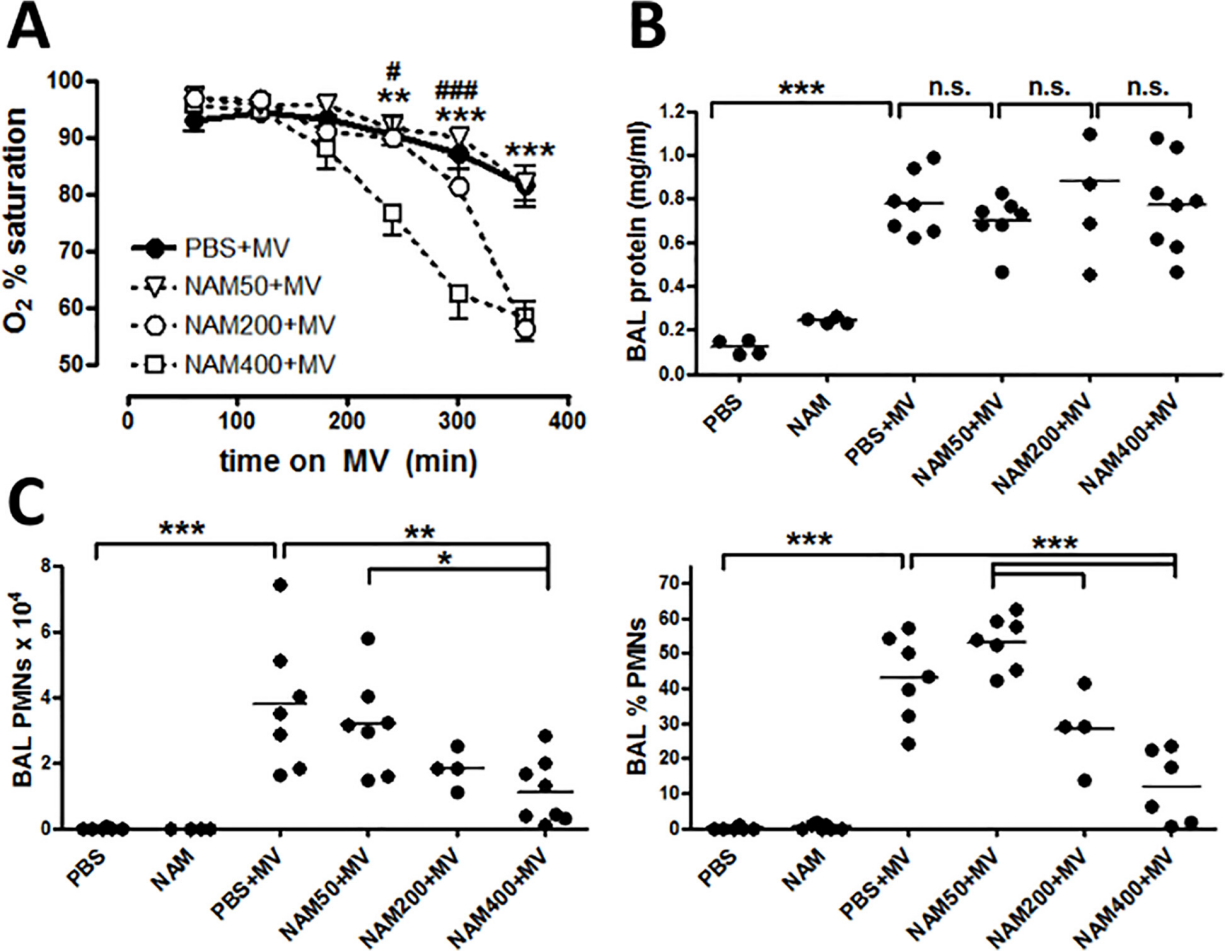
Effects of nicotinamide on hypoxemia and PMNs are dose-dependent. Mice were anesthetized and placed on mechanical ventilation as described in Fig 1. After one hour of mechanical ventilation, mice were injected intraperitoneally with PBS or NAM at doses of 50 mg/kg (NAM50+MV), 200 mg/kg (NAM200+MV) or 400 mg/kg (NAM400+MV). (A) Oxygen saturation was measured each hour. Significance is indicated for comparisons between NAM400 and NAM50 (** p < 0.01, *** p < 0.001) and between NAM400 and NAM200 (^#^ p < 0.05, ^###^ p < 0.001). Mice were euthanized at the end of 6 hours mechanical ventilation, and bronchoalveolar lavage (BAL) fluid was assayed for: (B) protein concentration and (C) neutrophil (PMNs) numbers and percentages of total BAL cells. PBS and NAM400 data are repeated from Fig 1 for comparison with NAM50 and NAM200. Data are representative of two separate experiments for NAM50 and NAM200, and four separate experiments for PBS and NAM400. Significance is indicated for comparisons between PBS and PBS+MV, and between PBS+MV and all NAM+MV doses. * p < 0.05, ** p < 0.01, *** p < 0.001.
