# Supplementary figures and images for: Nicotinamide Exacerbates Hypoxemia in Ventilator-Induced Lung Injury Independent of Neutrophil Infiltration
Source: PLoS One. 2015 Apr 13;10(4):e0123460. doi: 10.1371/journal.pone.0123460 (PMC4395431; doi:10.1371/journal.pone.0123460)

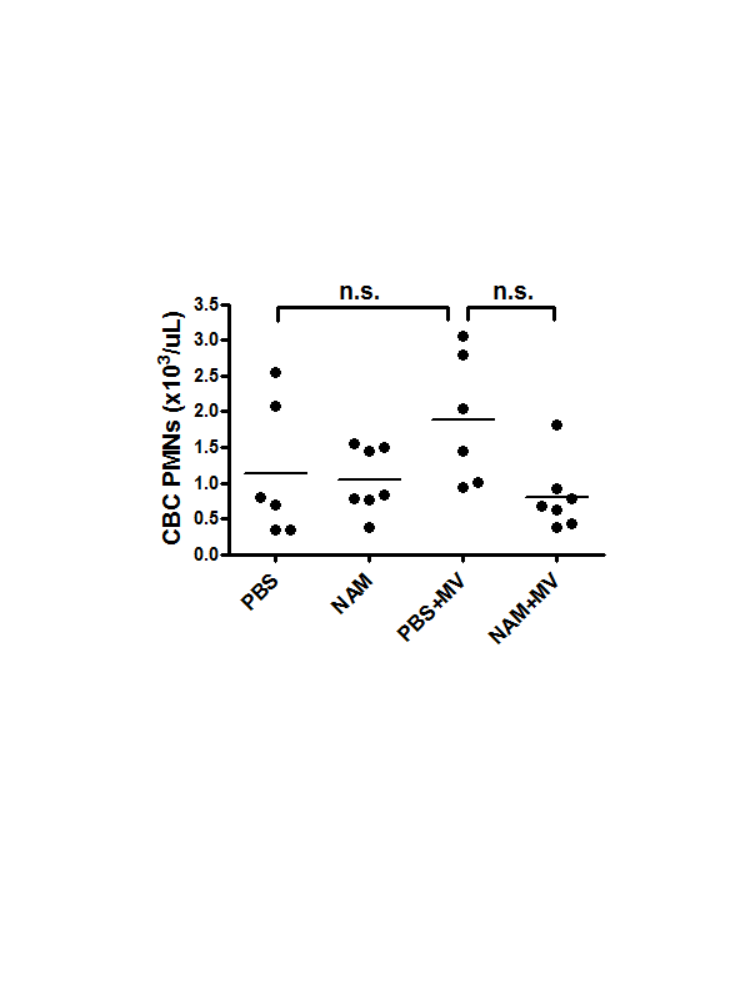

Supplement: S1 Fig — Mice were anesthetized and placed on mechanical ventilation (MV) with tidal volumes of 20 ml/kg and zero PEEP. After 1 hour of mechanical ventilation, mice received intraperitoneal injections of either 400 mg/kg nicotinamide (NAM+MV) or an equivalent volume of PBS (PBS+MV), and mechanical ventilation was continued for a total of 6 hours; control mice received intraperitoneal NAM or PBS at the same as the mice on mechanical ventilation (PBS, NAM) but were not anesthetized. All mice were euthanized at the end of 6 hours mechanical ventilation or spontaneous breathing (control mice). Whole blood was collected in syringes containing EDTA from the inferior vena cava, and analyzed for complete blood cincluding leukocyte counts and types of leukocytes (automated differential). Differences in neutrophil counts among groups did not reach statistical significance. n = 6-7/group. (TIF) [file pone.0123460.s001.tif]

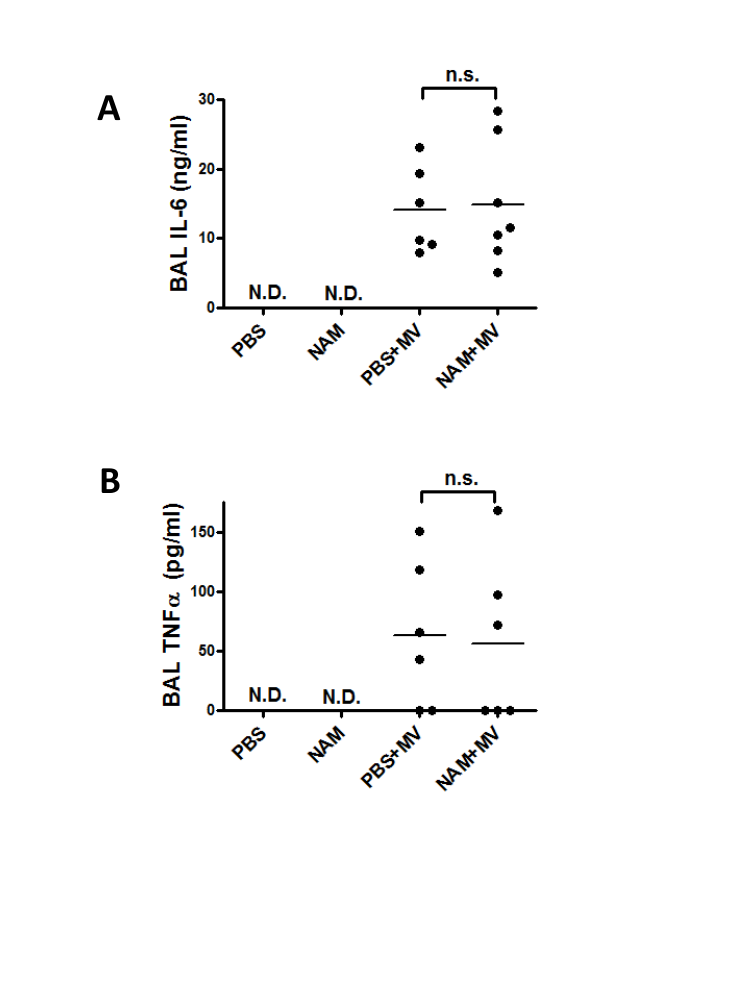

Supplement: S2 Fig — Mice were anesthetized and placed on mechanical ventilation (MV) with tidal volumes of 20 ml/kg and zero PEEP. After 1 hour of mechanical ventilation, mice received intraperitoneal injections of either 400 mg/kg nicotinamide (NAM+MV) or an equivalent volume of PBS (PBS+MV), and mechanical ventilation was continued for a total of 6 hours; control mice received intraperitoneal NAM or PBS at the same as the mice on mechanical ventilation (PBS, NAM) but were not anesthetized. All mice were euthanized at the end of 6 hours mechanical ventilation or spontaneous breathing (control mice), and bronchoalveolar lavage (BAL) fluid was assayed for: (A) IL-6 and (B) TNF. Differences in IL-6 and TNF concentrations between PBS+MV and NAM+MV groups did not reach statistical significance. N.D. signifies groups for which IL-6 and TNF concentrations were below the limit of detection. n = 6-7/group. (TIF) [file pone.0123460.s002.tif]

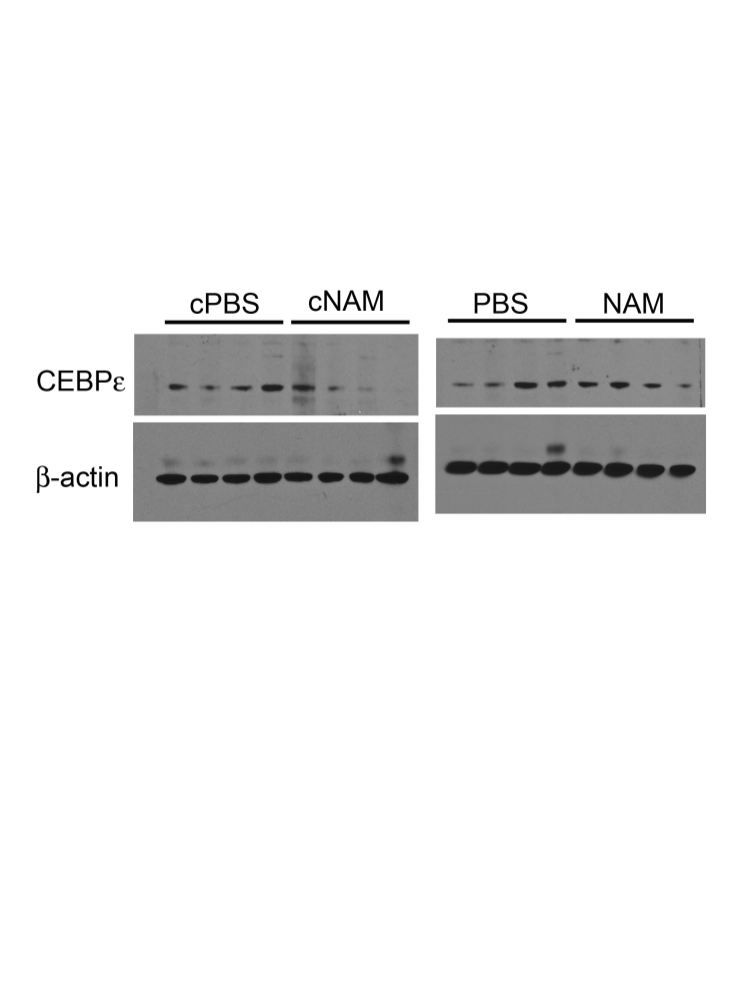

Supplement: S3 Fig — Mice were anesthetized and placed on mechanical ventilation (MV) with tidal volumes of 20 ml/kg and zero PEEP. After 1 hour of mechanical ventilation, mice received intraperitoneal injections of either 400 mg/kg nicotinamide (NAM) or an equivalent volume of PBS (PBS), and mechanical ventilation was continued for a total of 6 hours; control mice received intraperitoneal NAM or PBS at the same as the mice on mechanical ventilation (cPBS, cNAM) but were not anesthetized. All mice were euthanized at the end of 6 hours mechanical ventilation or spontaneous breathing (control mice). Whole lungs were homogenized in lysis buffer and analyzed via Western for C/EBPepsilon protein. (TIF) [file pone.0123460.s003.tif]
